# Psychological Status and Influencing Factors of Hospital Medical Staff During the COVID-19 Outbreak

**DOI:** 10.3389/fpsyg.2020.01841

**Published:** 2020-08-04

**Authors:** Yang Yao, Yao Tian, Jing Zhou, Xin Diao, Bogan Cao, Shuang Pan, Ligai Di, Yan Liu, Hui Chen, Chunxia Xie, Yuanli Yang, Feiyu Li, Yuqi Guo, Shengyu Wang

**Affiliations:** ^1^Department of Pulmonary and Critical Care Medicine, The First Affiliated Hospital of Xi’an Medical University, Xi’an, China; ^2^Department of Critical Care, Xi’an Chest Hospital, Xi’an, China; ^3^Department of Critical Care, The Eighth Hospital of Xi’an, Xi’an, China

**Keywords:** COVID-19, psychological status, health care workers, influencing factors, 12-item General Health Questionnaire (GHQ-12)

## Abstract

**Background:**

The aim of this study was to analyze the psychological status of and its influencing factors in health care workers (HCWs) during the coronavirus disease 2019 (COVID-19) outbreak so as to provide sufficient theory and scientific basis for the formulation and implementation of relevant policies and measures in improving the psychological status of HCWs.

**Method:**

During February 1 to February 20, 2020, 1,002 members of the HCWs from Xi’an and Wuhan completed a 12-item questionnaire regarding pressure about the COVID-19 influenza pandemic, along with the 12-item General Health Questionnaire (GHQ-12). The GHQ-12 scale was divided by three points. The positive group was scored more than 3. All data were analyzed by SPSS.

**Results:**

More than half of the participants (61.1%) reported psychological distress. The HCWs have sufficient information about the COVID-19 symptoms, prognosis, treatment, infection route, and preventive measures (medians ranged from 6/9 to 8/9). Female, engaged in clinic work less than 7 years, married person, and working in Wuhan were risk factors affecting the psychological status of HCWs (*P* < 0.05).

**Conclusion:**

Psychological distress is common in HCWs during the COVID-19 outbreak. Hospitals and relevant departments should provide psychological support to HCWs, and strict infection control measures should be developed.

## Introduction

Last December 2019, the epidemic of coronavirus disease (COVID-19) has a breakout in Wuhan, China (Paraskevis et al., 2020; Wang et al., 2020), and spread rapidly around the world. On February 19, 2020, WHO named the novel coronavirus pneumonia as “COVID-19,” and the new crown pneumonia is NCP. Early studies mainly focused on clinical characteristics, treatment measures, and epidemiological features (Chen N. et al., 2020; Chen Z.M. et al., 2020; Guan and Xian, 2020). In addition, this research paid more attention to patients but ignoring the health of health care workers (HCWs), especially psychological status. As of February 17, more than 32,000 HCWs from 220 medical teams across China have come to Hubei for support. However, the understanding of COVID-19-related knowledge and prevention measures is still in its infancy, coupled with the increase of workload and the risk of infection, resulting in a serious impact on the psychological health of HCWs. Moreover, the psychological state of HCWs is closely related to the therapeutic effect and prognosis of patients. Previous studies on the impact of disease outbreaks on the psychological health of HCWs have shown that many HCWs show a high degree of psychological distress, the pressure over severe acute respiratory syndrome (SARS) and Middle East respiratory syndrome (MERS) outbreak have been related to work pressure, social isolation, and health concerns (Lee et al., 2004; Wu et al., 2009). However, there are few studies have been conducted to investigate the psychological distress of HCWs at the height of the epidemic of COVID-19. This study aims to evaluate the psychological status of and influencing factors in HCWs during the outbreak of COVID-19 and to provide a scientific basis for improving the psychological status of the HCWs and making relevant policies.

## Materials and Methods

### Recruitment

The study was carried out by online survey from February 1 to February 24, 2020. A total of 1,002 respondents from The First Affiliated Hospital of Xi’an Medical University, Xi’an Chest Hospital, Xi’an Eighth Hospital, Wuhan Union Hospital (HCWs supported by Shaanxi), and Wuhan Ninth Hospital (HCWs supported by Shaanxi).

### Questionnaire Design

The questionnaire consists of three parts: basic characteristics, online survey, and the 12-item General Health Questionnaire (GHQ-12). The GHQ-12 has been widely used to assess mental health status (Montazeri et al., 2003).

(1) Basic Characteristics:
  Age, years of clinical work, marital status, education level, profession, and whether working in Wuhan were included in the basic characteristics.
(2) Online Surveys:

In order to reduce face-to-face communication and avoid infection, the existing research invites potential interviewees electronically. They completed the questionnaire in Chinese through the online survey platform (Surveystar, Changsha Ranxing Science and Technology, Shanghai, China). Previous surveys on the psychological impacts of SARS and influenza outbreaks were reviewed (Nickell et al., 2004; Goulia et al., 2010). Authors included additional questions related to the COVID-19 outbreak. Finally, this section contains 10 items which are all scored on a 9-point Likert scale, a higher score indicated a strongly degree.

(3) Twelve-Item General Health Questionnaire:

The questionnaire consists of 12 items, of which six are positive items and six are negative, which are scored at four levels. (1) never, (2) rarely, (3) sometimes, and (4) often. The most common scoring methods bimodal (0-0-1-1) are used according to WHO scoring method. If the answer is “often” or “sometimes,” it is 1 point. If the answer is “never” or “little,” it is 0 point. Results the higher the score, the lower the level of mental health. Therefore, this gives scores ranging from 0 to 12. The total score ≥3 was poor mental health.

### Statistical Analysis

The data were analyzed using SPSS software 18.0 (SPSS Inc., Chicago, IL, United States). Chi-square test or Fisher’s exact test was used for analysis of categorical variables. The ANOVA test or Student’s *t* test was utilized to compare measurement variables. *F* test was used if related to non-normal distribution parameters. Categorical variables were expressed as number (%) and mean (SD); logistic regression analysis was used to analyze the correlation before multiple factors. *P* < 0.05 was considered statistically significant.

## Results

### Basic Characteristics

The basic characteristics of the participants were shown in Table 1. The average age was 32.46 ± 6.86 years. Most of the participants were female (85.2%). Five hundred forty-nine (54.8%) participants engaged in clinical work for less than 7 years. For marital status, 68.8% were married, 30.7% were single, and 0.05% were divorced. For educational level, 22% were junior college, 55.4% were undergraduate, 21.4% were master, and 1.2% were doctors. For profession, 583 (58.2%) participants were nurses, 278 (27.8%) were doctors, 68 (6.8%) were technicians, and 72 (7.1%) were others. There are 702 (70%) participants work in Wuhan.

**TABLE 1:** Basic characteristics and psychological distress (*N* = 1,002).

Category	
**Age (years) [mean ± SD]**	32.46 ± 6.86
Female, *N* (%)	854 (85.2)
Male, *N* (%)	148 (14.8)
**Years of clinical work, *N* (%)**	
≤7	549 (54.8)
>7	453 (45.2)
**Marital status, *N* (%)**	
Married	689 (68.8)
Single	308 (30.7)
Divorced	5 (0.05)
**Education, *N* (%)**	
Junior college	221 (22)
Undergraduate	555 (55.4)
Master	214 (21.4)
Doctor	12 (1.2)
**Profession, *N* (%)**	
Nurse	583 (58.2)
Medical	279 (27.8)
Technician	68 (6.8)
Others	72 (7.1)
**Working in Wuhan, *N* (%)**	
Yes	702 (70)
No	300 (30)
GHQ-12 ≥ 3, *N* (%)	612 (61.1)
GHQ-12 < 3, *N* (%)	390 (38.9)

### The Distribution of Psychological Status

**FIGURE 1:**
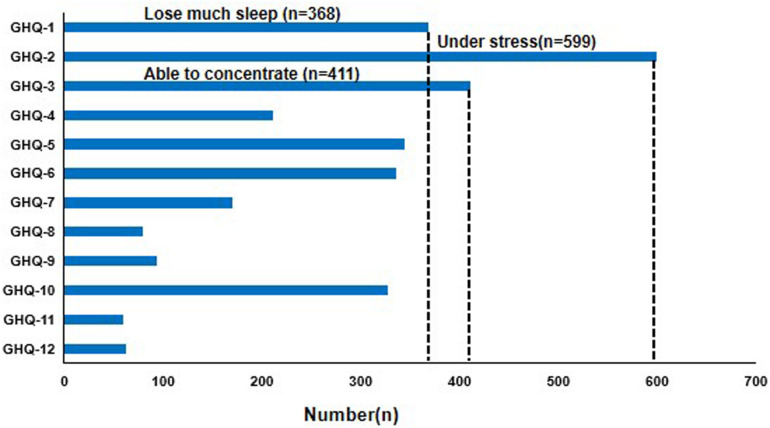
Number of people with a score of “1” for each item in the 12-item General Health Questionnaire (GHQ-12).

As shown in Figure 1, under stress (Item 2), able to concentrate (Item 3), and lose much sleep (Item 1) have the most participants with a score of 1. The numbers of participants were 599, 411, and 368, respectively. The major demographic, professional characteristics, and psychological distress scores were shown in Table 2. Six hundred twelve out of 1,002 participants (61.1%) presented scores on GHQ ≥ 3 (Positive), indicative of severe psychological distress. Three hundred ninety (38.9%) presented scores on GHQ < 3 (Negative), indicative of mild to moderate psychological distress. In the positive group, there were 512 (83.7%) females and 100 (16.3%) males. The difference of positive psychological stress between different genders was statistically significant (*P* = 0.007). Three hundred eight (50.3%) of the positive participants have engaged in clinical work for less than 7 years, and 304 (49.7%) participants had more than 7 years of work (*P* = 0.002). For marital status, 421 out of 612 positive participants (68.8%) were married, 189 (30.9%) were single, and 2 (0.3%) were divorced (*P* = 0.002). For educational level, 22.1% were junior college, 60.3% were undergraduate, 16.5% were master, and 1.1% were doctor (*P* = 0.001). For profession, 61.3% of positive participants were nurses, 24.8% were medical, 7.4% were technicians, and 6.5% were others (*P* < 0.001). Five hundred eighty-nine of 612 positive participants were working in Wuhan, and 23 were not (*P* = 0.049). There was no significant difference in age among the positive participants.

**TABLE 2:** The influence of demographic characteristics on the psychological stress of health care workers.

Category	GHQ ≥ 3 All = 612 (61.1%)	GHQ < 3 All = 390 (38.9%)	*P*-value
Age (years)			0.119
≤30	287 (46.9)	207 (53.1)	
>30	325 (53.1)	183 (46.9)	
Sex			0.007
Female	512 (83.7)	342 (87.7)	
Male	100 (16.3)	48 (12.3)	
Years of clinical work			0.002
≤7 years	308 (50.3)	241 (61.8)	
>7 years	304 (49.7)	149 (38.2)	
Marital status			
Married	421 (68.8)	268 (68.7)	0.002
Single	189 (30.9)	119 (30.5)	
Divorced	2 (0.3)	3 (0.8)	
Education			
Junior college	135 (22.1)	86 (22.1)	0.001
Undergraduate	369 (60.3)	186 (47.7)	
Master	101 (16.5)	113 (29.0)	
Doctor	7 (1.1)	5 (1.2)	
Profession			
Nurse	375 (61.3)	208 (53.3)	<0.001
Medical	152 (24.8)	127 (32.6)	
Technician	45 (7.4)	23 (5.9)	
Others	40 (6.5)	32 (8.2)	
Working in Wuhan			0.049
Yes	589 (96.2)	113 (29)	
No	23 (3.8)	277 (71)	

### Univariate Analysis of Factors Associated With the Psychological Distress of Health Care Workers

As shown in Table 3, we analyzed the source of psychological pressure according to occupations. The HCWs have sufficient information about the COVID-19 symptoms, prognosis, treatment, infection route, and preventive measures (medians ranged from 6/9 to 8/9). Medical staff scored the highest in terms of symptoms, prognosis, and treatment compared to the other groups. The difference is statistically significant (*P* < 0.001). Nurses have the highest score in infection route and preventive measures (*P* < 0.001). Most HCWs believe that their department provides clear information about COVID-19 (median 7/9). The score of nurse group was 6.87 ± 2.13, with the highest score compared with the other groups. Medical staff and technicians have confidence in the cure after infection. The scores were 4.71 ± 1.88 and 4.94 ± 1.91, respectively. While nurses and others were 5.17 ± 2.09 vs. 5.15 ± 2.41 (*P* < 0.001). The appeal for psychological support for medical staff is very high (median 9/9). But the differences between groups were not statistically significant. Most of the participants will not stop working during the outbreak of COVID-19 (median 2/9).

**TABLE 3:** Univariate analysis of factors associated with the psychological distress of health care workers (HCWs).

Category	Total (*N* = 1,002) (mean ± SD; median)	Medical (*N* = 279)	Nurse (*N* = 583)	Technician (*N* = 68)	Others (N = 72)	*P*-value
I believe that I have heard sufficient information about: (1: strongly disagree, 9: strongly agree)						
COVID-19 symptoms	7.18 ± 1.50, 7/9	7.27 ± 1.30	7.16 ± 1.52	7.32 ± 1.16	6.92 ± 2.19	<0.001
COVID-19 prognosis	6.61 ± 1.72, 7/9	6.75 ± 1.48	6.61 ± 1.77	6.22 ± 1.64	6.50 ± 2.19	<0.001
COVID-19 treatment	6.32 ± 1.83, 6/9	6.48 ± 1.61	6.34 ± 1.83	6.00 ± 1.80	6.32 ± 1.83	<0.001
COVID-19 infection route	7.66 ± 1.50, 8/9	7.64 ± 1.37	7.74 ± 1.51	7.51 ± 1.46	7.31 ± 1.82	0.001
COVID-19 preventive measures	7.57 ± 1.47, 8/9	7.52 ± 1.39	7.64 ± 1.47	7.47 ± 1.38	7.28 ± 1.83	0.016
I believe that my department provided clear information about the COVID-19 pandemic	6.73 ± 2.17, 7/9	6.53 ± 2.19	6.87 ± 2.13	6.21 ± 2.24	6.86 ± 2.28	0.023
Infection has a great impact on my health	5.63 ± 2.32, 5/9	5.42 ± 2.23	5.71 ± 2.33	5.96 ± 2.28	5.43 ± 2.59	0.303
It’s hard to cure after infection	5.02 ± 2.06, 5/9	4.71 ± 1.88	5.17 ± 2.09	4.94 ± 1.91	5.15 ± 2.41	<0.001
Would you avoid going to work	3.11 ± 2.60, 2/9	3.24 ± 2.56	3.01 ± 2.63	2.88 ± 2.32	3.69 ± 2.59	0.314
Should psychological support be given to HCWs	7.53 ± 1.98, 9/9	7.23 ± 2.09	7.67 ± 1.88	7.65 ± 2.09	7.46 ± 2.11	0.702

### Logistic Analysis of Factors Associated With the Psychological Distress of Health Care Workers

As shown in Table 4, female, engaged in clinic work less than 7 years, married person, and working in Wuhan were risk factors for the psychological status of HCWs. Females suffer 2.561 times psychological distress more than men [odds ratio (OR) = 2.561, 95% CI 2.435–2.778]. The psychological distress of clinical work less than 7 years is 2.669 times than that of work more than 7 years (OR = 2.669, 95% CI 2.513–2.886). Married people are 1.639 times than single (OR = 1.639, 95% CI 1.355–1.922). HCWs working in Wuhan is 3.005 times than that working in other provinces (OR = 3.005, 95% CI 1.185–7.623). The difference is statistically significant (*P* < 0.001).

**TABLE 4:** Logistic regression analysis of related factors.

Category	OR	OR 95% CI	*p*-Value
**Sex**	2.561	2.435–2.778	0.021
**Age**	1.208	0.797–1.830	0.373
**Years of clinic work**	2.669	2.513–2.886	0.042
**Marital status**			0.024
Married			
Single	1.639	1.355–1.922	0.014
Divorced	1.864	0.283–12.259	0.517
**Education**			0.785
College			
Undergraduate	0.581	0.159–2.123	0.411
Master	0.676	0.192–2.375	0.541
Doctor	0.713	0.207–2.447	0.592
**Profession**			0.011
Nurse			
Medical	0.615	0.300–1.261	0.184
Technician	1.467	0.805–2.676	0.211
Others	0.937	0.435–2.017	0.868
**Working in Wuhan**	3.005	1.185–7.623	0.021
**COVID-19 symptoms**	1.060	0.912–1.232	0.451
**COVID-19 prognosis**	0.916	0.799–1.049	0.204
**COVID-19 treatment**	1.075	0.960–1.205	0.210
**COVID-19 infection route**	1.007	0.857–1.184	0.931
**COVID-19 preventive measures**	0.958	0.808–1.137	0.627
**I believe that my department provided clear information about the COVID-19 pandemic**	1.041	0.779–1.390	0.788
**Infection has a great impact on my health**	0.584	0.435–0.785	0.000
**It’s hard to cure after infection**	0.772	0.564–1.055	0.105
**Would you avoid going to work**	0.999	0.945–1.055	0.966
**Should psychological support be given to HCW**	1.134	1.054–1.220	0.001

## Discussion

Hospital medical staff show an absolutely important position in the outbreak of infectious diseases, but people often pay more attention to the cure rate, diagnosis, and treatment effect and prognosis of patients and ignore the psychological distress of HCWs. Studies that investigated the psychological status during SARS and A/H1N1 influenza pandemic indicated that a high level of distress is common (Caputo et al., 2006; Goulia et al., 2010). In addition, we are dealing with an epidemic the likes of which we have never seen in this century. As of February 25, 2020, 77,779 confirmed and 2,688 death cases have been reported in China and spread rapidly in 34 Chinese provinces or municipalities. Therefore, it is urgent to investigate the psychological state of and related factors in medical staff and provide a certain scientific basis for improving the psychological status of HCWs. Our results showed that during the period of the COVID-19 epidemic, more than half of HCW participants have suffered from psychological distress. The proportion of married female is relatively large. It may be related to women’s physiological reasons. Compared with men, their ability to bear pressure is slightly weaker. The main reason for married people’s stress is that their work increases the risk of infection among family members. The score of physiological distress of nurses was higher than that of other staff. Although both doctors and nurses are in contact with patients, medical staff expressed a lower degree of psychological distress. The possible reason may be that medical staff mostly regarded themselves as sufficiently informed, and it is generally true that medical staff are highly educated and have sufficient knowledge reserve, so they have a better understanding of the information they have acquired. In addition, nurses are the largest occupational group in the hospital. They have a direct and close relationship with patients and have a higher risk of infection. Therefore, it is easy to understand that nurses think themselves have a full understanding of the infection pathway and prevention measures of COVID-19 and have the highest voice for psychological support. The study also found that long-term clinical work can reduce psychological stress. This may be due to that the rich experience can be accumulated through long-term work, which can better cope with emergencies and complex situations. Finally, the psychological distress of medical staff working in Wuhan is higher than that out of Wuhan. The COVID-19 outbreak is in Wuhan, and until now, Wuhan is still the worst-hit region of COVID-19 infection. The number of confirmed cases and mortality rate in there have been ranked first in the country, accounting for 96% of total mortality rate. In addition, the disease has been confirmed to be human–human transmission (Du et al., 2020; Xu and Kraemer, 2020). According to reports, more than 3,000 medical staff have been infected with COVID-19. In addition, most of the medical staff are non-native. Unfamiliarity with the local environment and language increases their psychological pressure. These results are consistent with the results of previous studies in SARS. Angelina OM Chan performed a study focused on psychological impact of the 2003 SARS outbreak on HCWs in Singapore and found that 27% participants had psychological distress (Chan and Huak, 2004). Bai et al. (2004) also investigated stress reactions among 338 staff members in a hospital in East Taiwan and came to the conclusion that 5% suffered from an acute stress disorder during the SARS outbreak.

As the COVID-19 epidemic continues to spread, our findings will provide important guidance for the development of psychological support strategies for China and other affected areas. Our findings also have clinical and policy implications. The results show that female medical staff are suffering from greater psychological impact and higher levels of pressure in this epidemic. This will help health authorities to identify high-risk groups for early psychological intervention. Moreover, as concerns about protective measures are a major source of stress, strict and detailed infection control measures should be developed. Furthermore, to minimize face-to-face interaction, health authorities may consider providing online or smartphone-based psychological education and intervention.

This study also has several limitations. Firstly, there is gender bias in this study. Comparison of the characteristics of the research samples in this epidemic suggested that the study sample is gender-specific. Despite this, however, we cannot refute the criticism that an underlying response style might have led to our results. Secondly, there is no classified analysis on the psychological status of medical staff in the intensive care unit (ICU) and non-ICU, which may also be a potential factor. Finally, social discrimination is ignored in this study.

## Conclusion

There is a general psychological pressure among medical staff during the COVID-19 pandemic. Pressure was significantly associated with preventive measures and infection route. The perceived sufficiency of information and psychological intervention measures about COVID-19 was associated with a reduced degree of pressure.

## Data Availability Statement

All datasets presented in this study are included in the article/supplementary material.

## Ethics Statement

Ethical review and approval was not required for the study on human participants in accordance with the local legislation and institutional requirements. Participation was anonymous via an online survey questionnaire.

## Author Contributions

YuY and YT designed the study and drafted the work. JZ and XD conducted the analysis. BC made substantial contribution to the design of the work. SP and LD contributed to the interpretation of the work and revising the draft for important intellectual content. YL, CX, and HC helped with access to the data and provided information and consulting. SW and FL made substantial contributions to the conception of the work. YG and SW revised the draft for important intellectual content and agreed to be accountable for all aspects of the work in ensuring that questions related to the accuracy or integrity of any part of the work are appropriately investigated and resolved. All authors reviewed and approved the final manuscript.

## Conflict of Interest

The authors declare that the research was conducted in the absence of any commercial or financial relationships that could be construed as a potential conflict of interest.
